# Disseminated Paracoccidioidomycosis in a Kidney Transplant Recipient

**DOI:** 10.7759/cureus.19007

**Published:** 2021-10-24

**Authors:** Carlos Rafael A Felipe, Aline D Silva, Maria Goretti Moreira Guimarães Penido

**Affiliations:** 1 Nephrology Center, Santa Casa de Belo Horizonte Hospital, Belo Horizonte, BRA

**Keywords:** kidney transplantation, itraconazole, immunosuppression, blastomycosis, paracoccidioidomycosis

## Abstract

Paracoccidioidomycosis (PCM) is an endemic fungal infection in Latin America, which manifests as an acute or chronic form and is more frequent in adult males. It is caused by *Paracoccidioides brasiliensis* or *Paracoccidioides lutzii*, which are thermodimorphic fungi. The disease can present as a severe and disseminated form involving the lungs, skin, lymph nodes, spleen, liver, and lymphoid organs of the gastrointestinal tract. Most of the primary infections are subclinical, and the cell-mediated immune response contains the infection. It is rare in transplant patients, and there are few cases described in the literature. In solid organ transplant patients, it usually results from the reactivation of a latent infection, manifesting itself after a few years of transplantation with frequent pulmonary and skin involvement. PCM is an endemic infection in Brazil; however, as it is not classified as a notifiable disease, there is no accurate database on its incidence, and case reports are important sources of information. Clinical disease in kidney transplant patients is rare and has a high mortality rate. In this scope, the present clinical case reports the challenges of the clinical management of disseminated PCM caused by *Paracoccidioides brasiliensis* in a kidney transplant recipient who used immunosuppressive drugs and was treated with Itraconazole.

## Introduction

Paracoccidioidomycosis (PCM) is a disease caused by dimorphic fungi such as* Paracoccidioides brasiliensis* and *Paracoccidioides lutzii*. It is common in Latin America, and almost 80% of cases occur in Brazil [1,2]. Most cases are asymptomatic, but men are 10-13 times more likely to have symptoms than women [1,2]. There are two clinical forms of PCM: acute/subacute and chronic. The acute/subacute form is more common in children and young adults and presents with fever, lymphadenopathy, hepatosplenomegaly, anemia, eosinophilia, and weight loss [2,3]. Cutaneous, bone, and pulmonary involvements are limited or absent [4,5]. The chronic form represents 80%-90% of cases and manifests with frequent involvement of the lungs, skin, and mucous membranes [5].

The gold standard diagnostic test identifies the fungus by direct examination, culture, and histopathology of the affected tissues. The detection of specific antibodies against parasite antigens and specific polymerase chain reaction (PCR) assays are other valuable testing methods [2,6]. The treatment of mild/moderate cases consists of itraconazole alone or with sulfamethoxazole-trimethoprim (SMX-TMP) [7]. Disseminated infections require the use of amphotericin B, followed by consolidation therapy with itraconazole, and the duration varies from 12 to 24 months, depending on the clinical presentation [7,8].

Evidence-based information on PCM in immunocompromised patients is scarce despite the growing number of kidney transplantation (KTx) cases in endemic areas. PCM in these patients has emerged as a late chronic infection years after KTx, ranging from five to 14 years [9]. We present a case that illustrates the challenges in clinically managing disseminated PMC infection in an immunosuppressed KTx recipient treated with itraconazole.

## Case presentation

A 47-year-old male, born in Pedro Leopoldo City, Minas Gerais, Brazil, lost kidney function due to immunoglobulin A (IgA) nephropathy and underwent a second KTx at Santa Casa de Belo Horizonte Hospital (SCBH) in 2015 with a kidney from a related living donor (his first KTx occurred in 2011 and was unsuccessful due to chronic rejection). The second transplant was complicated with polyomavirus nephropathy and calcineurin inhibitor nephrotoxicity. His immunosuppression regimen consisted of tacrolimus, everolimus, and prednisone.

**Figure 1:**
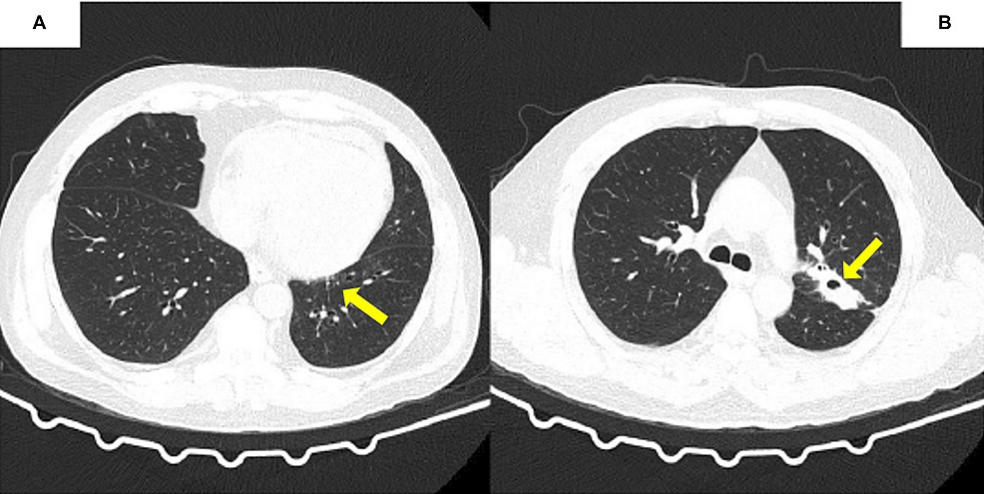
High-resolution computed tomography scan of the chest showing consolidation (A) and an excavated nodule, with thickened walls and inverted halo sign (B).

**Figure 2:**
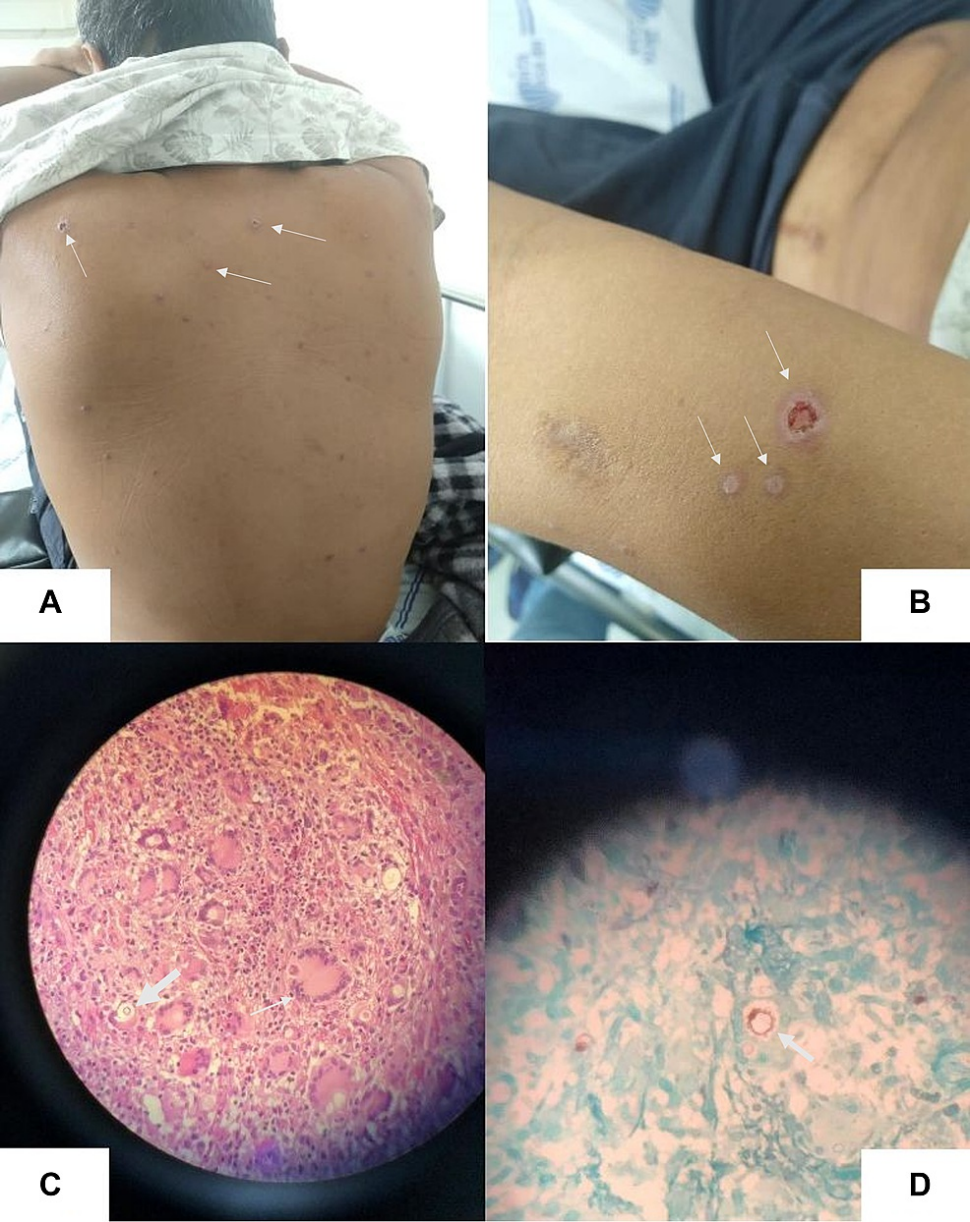
Rounded cutaneous ulcerated lesions with erythematous fundus and elevated border, disseminated in the dorsal region (A) and upper limbs (B). Histopathology shows granulomatous inflammatory response characterized by macrophages, Langhans type multinucleated giant cells (thin arrow), and isolated and grouped fungi phagosomes (thick arrow) (C), confirming the presence of Paracoccidioides brasiliensis. Rudder wheel shape of the parasite (D).

The patient came to the emergency room with a dry cough, shortness of breath, and chills. He reported a one-month weight loss of 10 kg. Examination on admission showed anemia (Hb: 8.8 g/dL; Hct: 29.2%), elevated C-reactive protein levels (46.3 mg/dL (reference range: <5 mg/dL)), and impaired renal function (SCr: 242.2 umol/L (reference range: 70-105 umol/L)). He reported shortness of breath, even while resting, and he was referred to the SCBH. Rapid serological testing for coronavirus disease 2019 was nonreactive. Three sputum samples were negative for acid-fast bacilli (Ziehl-Neelsen staining). His chest computed tomography scan showed a solid lesion involving the left upper lobar bronchus associated with consolidation and an area of excavation with thickened walls, suggesting tuberculosis or malignant neoplasia (Figure 1). Fiberoptic bronchoscopy was conducted to collect bronchoalveolar lavage (BAL) and transbronchial lung biopsy (TBLB). PCR of the material was negative for tuberculosis, the direct test was negative for malignant cells, and fungi and culture showed no bacterial growth. TBLB material showed fungal structures compatible with cryptococcosis, blastomycosis, or PCM (not shown). We also observed concomitant disseminated painless, erythematous, rounded skin lesions with raised edges and central ulceration, and a skin biopsy was performed (Figure 2). The review of the BAL, TBLB, and skin biopsy contributed to the diagnosis of PCM.

Considering the possibility of worsening renal function and the need for dialysis, the patient’s care team decided to start itraconazole. Subsequent evaluations revealed elevated liver and canalicular enzymes (ALP: 848 U/L (reference range: 65-300 U/L); GGT: 1547 U/L (reference range: 12-64 U/L); AST: 141 U/L (reference range: 5-34 U/L); ALT: 111 U/L (reference range: <55 U/L)) with a predominance of direct hyperbilirubinemia (direct bilirubin: 2.7 mg/dL (reference range: <0.2 mg/dL); indirect bilirubin: 0.1 mg/dL (reference range: 0.1-1.0 mg/dL)) and progressive worsening of renal function (SCr: 422.6 umol/L (reference range: 70-105 umol/L)). Tacrolimus was discontinued, and a renal biopsy was performed. We only identified a histological recurrence of IgA nephropathy with no signs of fungal structures or inflammation in the renal parenchyma. Leukocytosis and eosinophilia were never observed.

The patient showed progressive clinical and laboratory improvement. After 49 days of hospitalization, he was discharged using itraconazole with proposed maintenance therapy for at least 12 months. The only immunosuppressant maintained was everolimus. He is currently monitored via follow-up at the SCBH.

 

## Discussion

PCM in KTx recipients is rare, but its chronic form has been described in the literature [10-12]. Almeida et al. reported that their patients had a median age of 55 years; most were males and developed PCM between one and 14 years after KTx [5]. Our patient was a 47-year-old male, and PCM was diagnosed five years after the second transplant. In our case, the time between transplantation and clinical fungal infection is shorter than what is reported in the literature; however, it is still classified as a late complication of transplantation.

The clinical manifestations of chronic PMC vary, and clinical and radiological pulmonary involvement is frequent [10,13]. Góes et al. described the case of a 66-year-old male who developed PCM after one year and seven months of KTx and presented unusual skin lesions [2]. Pontes et al. described the case of a patient without pulmonary concerns who developed PCM shortly after KTx, with diarrhea and rapid deterioration of his general condition [10]. Our patient progressed with multisystem involvement characterized by a cavitary lung lesion, disseminated ulcerated skin lesions, elevated liver and canalicular enzymes, and direct hyperbilirubinemia. Severe acute kidney injury was also identified, although the kidney biopsy was not able to prove direct invasion of kidney tissue.

No radiological findings are pathognomonic in PCM. Bilateral diffuse mixed interstitial and alveolar infiltrate are the most common findings. Cavitation is rare, but when present, they are small, single or multiple, and located in the central areas of the lungs [6,11,14]. Our patient’s case is especially rare due to its unusual radiological presentation.

PCM diagnosis is based on clinical signs and symptoms, direct microscopic examination of specimens, isolation of fungi in culture, and detection of specific antibodies by serological techniques [6,11]. Skin scrapings and biopsy of the affected organ can also be included in PCM diagnosis [6,11]. Our patient was diagnosed with BAL, skin lesions, and TBLB.

The standard treatment for mild/moderate forms of PCM is itraconazole [7,8]. Voriconazole shows similar efficacy, and SMX-TMP is an alternative treatment [7,8]. Patients with severe and disseminated forms of PCM use liposomal amphotericin B, and physicians should consider corticosteroids [7,8]. Therapeutic regimens with other antifungal agents, such as amphotericin B deoxycholate and terbinafine, were evaluated as therapeutic options [3,8]. Our patient was in good clinical condition and received itraconazole to minimize nephrotoxicity and preserve the graft.

Patients receiving a second KTx are at high immunological risk, and management with triple-therapy immunosuppression is important to prevent graft rejection and loss. The state of intense immunosuppression compromises the innate/adaptive immune response against pathogens and makes the control of patients with PCM even more challenging [1,5]. Further challenges in managing PCM are the long treatment period and the high frequency of relapses and sequelae. However, vaccines may offer a promising strategy. A study with peptide P10 tested in an animal model induced an immune response, reducing the fungus load in immunosuppressed patients [15].

There is no report in the literature of direct transmission of PCM in KTx, and there is no formal recommendation to screen PCM in the living donor or recipient for prior contact/latent infection before the KTx [5,12]. However, as this case report highlights, physicians should still consider the rare possibility that the donor can transmit PCM during the living KTx, and the condition can remain latent until clinical presentation five years later.

## Conclusions

PCM in KTx recipients appears to have a poor prognosis related to low clinical suspicion, difficult and late diagnosis, and immunosuppression. PCM should be considered in the differential diagnosis of infections in the first year of transplantation (especially with pulmonary or cutaneous involvement) for patients in endemic areas. Nephrologists must always be aware of this diagnostic possibility because appropriate early treatment can positively affect patient outcomes and preserve the graft function.
